# Is there any difference based on sublingual varices frequency between hypertensive patients and healthy persons?

**DOI:** 10.1186/s12903-023-03396-y

**Published:** 2023-09-15

**Authors:** Hakimeh Ahadian, Mohammad Hasan Akhavankarbassi, Yasaman Sabaghzadegan, Fatemeh Owlia, Amir Sasha Daneshmand

**Affiliations:** 1 Department of Oral and Maxillofacial Medicine, School of Dentistry, Shahid Sadoughi University of Medical Sciences, Yazd, Iran; 2 International Campus, School of Dentistry, Shahid Sadoughi University of Medical Sciences, Yazd, Iran

**Keywords:** Blood pressure, Hypertension, Sublingual varices, Tongue

## Abstract

**Background:**

Sublingual varices (SV) are benign vascular lesions that have questionable associations with aging, smoking, and hypertension. This study purposed to evaluate whether SV frequency differs between hypertensive patients and healthy persons.

**Methods and Material:**

This cross-sectional study was conducted on 120 dental patients referred to Yazd Oral Medicine Department. At first sublingual surface of the tongue were examined before the routine oral survey, and blood pressure (BP) was measured in a sitting position. SV were classified into Grade 1 (no/mild), Grade 2 (medium to severe), and Grade 3 (multi-focal). Blood pressure was measured in all participants. Participants based on the obtained BP, divided into normal, prehypertension, stage 1, and 2 groups. Frequency of SV was compared in regarding age, gender, and stage of hypertension. All of the analyses were conducted at a *p* < 0.05 level of significance by the SPSS22 statistical package using chi-square test.

**Results:**

Out of 120 patients, 84 (70%) had SV. The frequency of SV in patients with hypertension (HT) (86.8%) was significantly higher than in others (48.1%); (*p* < 0.001). Data analysis showed there was a significant difference between the age groups. There was no significant difference based on SV between two genders. There was no significant difference in the frequency of sublingual varices between males and females. Aging could impact the frequency of SV.

**Conclusion:**

This study revealed that patients with hypertension had more SV. A simple valuable method for dental clinicians to be active in preventive health care is evaluating sublingual surface of tongue.

## Introduction

### Background

Varicosities are acquired vascular abnormalities which determined as locally increasing the size of the vein’s lumen. Varicosities may be seen in the oral cavity, uncommonly. The most common locations in oral cavities are sublingual, buccal mucosa, retro commissural mucosa. The sublingual area is important in the success of the implant due to its proximity to the alveolar ridge [1]. They appeared dark blue or purple with positive diascopy [2–4]. The lesions are usually asymptomatic and may be detected during routine clinical examination [5].

Sublingual varicosity (SV), at first, was described by DaCosta in 1930 and later was described with named caviar tongue. Some etiologic factors such as connective tissue and venous structure disorders have been suggested [6, 7]. The prevalence of SV in general population has a range of 1.5 to 16.2% [7–9]. Jafari reported prevalence of SV in Iranian nursing homes is 56.7% [10]. Some related factors could impact the incidence of SV. Age is a known risk factor for sublingual varices and the prevalence of this has been reported in 11 to 60% of elderly [11–13]. Al-shayyab declared that SV could be associated with abnormalities in the circulation system that occurred in specific disease [7]. Some studies have suggested the relationship between SV and heart disease. Smoking and use of dentures have a potent role in increasing the rate of sublingual varices [4, 14–17]. Hedström reported that identifying first-degree sublingual varices has led discovery of 48% of patients with high-grade hypertension. According to the results, 50% of patients older than 40 years with sublingual varices had high blood pressure [18].

The role of high blood pressure in the development of sublingual varicose veins has been studied for a long time. The dentist can play a significant role in the early diagnosis of high blood pressure by carefully examining the tissues of the tongue and under the tongue, and acquaintance with common oral lesions in the elderly can be very helpful for dentists and can prevent further unreasonable treatments [17].

This study purposed to evaluate whether SV frequency differs between hypertensive patients and healthy persons.

## Methods

### Design and setting

This cross-sectional study was performed at the Yazd oral medicine department.

### Study population

The sample size was calculated according to one proportion estimation formula. Sampling by census method among those who refer to the Department of Oral Medicine considering the confidence level of 95% power 80%, and 50% prevalence of high blood pressure based on a pilot conducted among the clients of the Department of Oral Medicine in people without varicose veins and people with varicose veins. The minimum required sample size was estimated to be 60 cases. In order to increase reliability, 120 people were included in the study.

Patients above the age of 18 years were requested to take part in the study in connection with their regular visits. Those who accepted to participate provided written consent. Exclusion criteria were pregnancy, atrial fibrillation, renal disease, and smoking [18]. This research was presented to the ethics committee of Shahid Sadoughi University of Medical Sciences and approved the study with number IR.SSU.REC.1396.22.

### Procedure

Information about age, gender, medical and dental history, and social habits of participants were recorded on the related checklist. At first sublingual surface of the tongue was examined before the routine oral survey. Patients rested for at least 5 min in a quiet room. Blood pressure was measured in a sitting position. The blood pressure of all participants was measured by a Mercury -pressure gauge (YAMASU 600, Japan) at two standard intervals of at least 20 min [19].

In this study blood pressure of all participants was taken by an oral medicine specialist. Considering the nature of dentistry, it is enough to know the stage of hypertension of patients instead of exact quality. All patients placed in one of four groups according to the JNC7 classification [4]. An average systolic blood pressure (SBP) ranging from 140 to 159 mm Hg and or a diastolic blood pressure (DBP) ranging from 90 to 99 mm Hg was classified as stage 1 hypertension, and an average SBP of 160 mm Hg or higher and or a DBP of 100 mm Hg or higher as stage 2. Patients were evaluated for sublingual varices. Varices have been categorized into three degrees including first degree (no / mild), second degree (moderate to severe), and grade three (multi-focal) [19].

### Statistical analysis

The variables from the checklist were coded. All statistical analyses were performed using IBM SPSS 22 using Chi-square test. strength of the association between hypertension and sublingual varices was assessed. All tests were two-sided and the significance cut-off was set at 0.05.

## Result

A total number of 120 patients participated in the study. The age range of the participants was between 18 to 79 years (mean age 45.2 ± 15.1). The gender distribution was 55 males and 65 females of the subjects, 52 (43.3%) patients had normal BP and 68 (56.7%) had high BP. According to Table 1, There was no significant difference based on SV between two genders. There was a significant relationship between the higher age with increasing sublingual varices.

**Table 1:** Comparison of frequency distribution of sublingual varices according to gender and age groups

	Number	SV		*p*-value
		Without	with	
**Gender**				
Male	55	18 (32.7)	37 (67.3)	0.549^a^
female	65	18 (27.7)	47 (72.3)	
**Age groups**				
< 30 yrs	27	17 (63)	10 (37)	< 0.001^a^
30–50 yrs	44	13 (29.5)	31 (70.5)	
> 50 yrs	49	6 (12.2)	43 (87.8)	

^a^Chi square

In Table 2, a statistically significant difference was observed in terms of frequency of SV between the investigated groups.

**Table 2:** Comparison of the frequency distribution of SV based BP staging

BP	Number	SV			*P* value
		Without	grade 1	grade 2	
Without	52	27 (51.9%)	25 (48.1%)	0	
Pre HT	52	8 (15.4%)	44 (84.6%)	0	< 0.001^a^
Stage 1,2	16	1 (6.3%)	12 (75%)	3 (18.8%)	

^a^Chi square

## Discussion

These findings showed that the frequency of sublingual varices in hypertensive individuals was approximately twice as high as in those with normal blood pressure. SV as an indicator of risk for hypertension should be evaluated.

Dentists are among the first healthcare groups that may confront the first sign and symptom of systemic disease and play an important role in timely diagnosis and better management of it.

SV is a common benign clinical finding that could be related to high blood pressure, it is useful to screen people for identifying the condition of hypertension and timely treatment. It may help in the early diagnosis of patients with hypertension after the detection of sublingual varices. This study is designed to assess the possibility of this relationship.

It is worth mentioning that a case–control study should be done based on a minute estimate of past epidemiological studies on similar populations, therefor due to the loss of such studies on our society, our study was designed as an observational clinical study.

Our study was similar to other studies that selected their target group from patients who attended the dental center [7, 18, 19]. However, there are studies that have queried their data from their archives [13]. In our study, all participants were examined for SV by a well-educated last year dental student and oral medicine specialist who monitored suspicious lesions. Patients younger than 12 years old did not enter the study like Al-shayab et al. as they were in the pediatric group and also due to the likelihood of traumatic injuries [7].

The present study showed that the frequency of SV was 70%. This percentage is higher than the range of past studies which reported 2.7 to 41.1%0.7 [13, 18, 20–22]. It seems that the difference between the results of various studies may be due to the intrinsic differences between populations, the sampling method, age factor, or diagnostic indices.

In confirmation of these cases, Rabiei et al. Reported the prevalence of sublingual varicosity in the elderly of Rasht by 22.7% [23]. While Mansour Ghanaei et al. Examined the prevalence of varicosity in Rasht, with a population of 30 and older, 1.5% [9]. Differences in the sampling method and age of study groups lead to inconsistency of results of those studies in the same geographic location.

According to our findings, there is no significant difference between two genders in the occurrence of sublingual varices. It is in accordance with Nevalainen et al. who reported similar results about sublingual varices [22]. But Corrêa et al. Revealed a higher incidence of sublingual varices among women than men [6]. Al-Shayyab and Baqain also identified females as a risk factor for sublingual varices [7].

In some literature, diagnosis of hypertension was not verified by blood pressure measurement, based on what was self-reported [24], but in our study an oral medicine specialist did it.

According to the discrepancy between the results of various studies, it is necessary to evaluate the relationship between sublingual varices and gender in studies with larger sample sizes. The present study showed that the mean age of patients with sublingual varices was significantly higher than others and with increasing age, the incidence of oral varicosity increased (Table 1). These results go in parallel with the conclusions of other literature [15, 25, 26].

This positive relation could be explained by the degeneration of vascular tissue elasticity with aging. Sublingual varices lesions may be considered as a physiological change associated with aging [8].

Based on the results of this study, the frequency of SV in patients with hypertension was significantly higher than in healthy persons. In addition, in hypertensive individuals, the frequency of SV increased with hypertension staging. This subject wasn’t discussed before. This finding confirms the results of Hedström study found a significant correlation between the incidence of sublingual varices and hypertension [18]. Al-Shayyab and Baqain also identified cardiovascular disease as a risk factor for underlying varicosity [7].

Hedström et al. Also found that the mean SBP and DBP in patients with grade 1 varices were higher than those with grade 0 varices [19].

The relationship between hypertension and SV could be explained based on tissue characteristics of the thin lining of the ventral surface tongue. Connective tissue network has an unstable structure in this area, therefor various factors such as hypertension may lead to dilatation and vascular varicosity changes in this area. An exact examination of the oral cavity by the oral clinician to detect sublingual varices with no harm to the patient could be a valuable method to early diagnosis of hypertension.

## Limitations

We are aware that our research suffers a number of limitations. The most important limitation lies in cooperating in examination for elderly patients was difficult to some extent. It is vital to be aware of the predictive limitations of cross-sectional studies, and a causal relationship can be clarified by carrying out additional prospective research. There is clearly much room for further research in this respect.

## Conclusion

The findings of this study showed that the frequency of sublingual varices in hypertensive individuals was approximately twice as high as in those with normal blood pressure. Aging could impact the frequency of SV. Examining the sublingual area as a simple valuable method of timely diagnosis and referral of probable disease would be suggested.

## Data Availability

The datasets analyzed during the current study are available from the corresponding author on reasonable request**.**

## Abbreviations

SV: Sublingual varices
BP: Blood pressure
SBP: Systolic blood pressure
DBP: Diastolic blood pressure
